# Acute MUS81 depletion leads to replication fork slowing and a constitutive DNA damage response

**DOI:** 10.18632/oncotarget.5497

**Published:** 2015-09-22

**Authors:** Meichun Xing, Xiaohui Wang, Timea Palmai-Pallag, Huahao Shen, Thomas Helleday, Ian D. Hickson, Songmin Ying

**Affiliations:** ^1^ Department of Pharmacology, Zhejiang University School of Medicine, Hangzhou, China; ^2^ School of Life Sciences, University of Lincoln, Lincoln, United Kingdom; ^3^ Department of Respiratory and Critical Care Medicine, Second Affiliated Hospital, Institute of Respiratory Diseases, Zhejiang University School of Medicine, Hangzhou, China; ^4^ State Key Laboratory For Respiratory Diseases, Guangzhou, China; ^5^ Science for Life Laboratory, Division of Translational Medicine and Chemical Biology, Department of Medical Biochemistry and Biophysics, Karolinska Institutet, Stockholm, Sweden; ^6^ Center for Chromosome Stability and Nordea Center for Healthy Aging, Department of Cellular and Molecular Medicine, University of Copenhagen, Copenhagen, Denmark

**Keywords:** cellular senescence, DNA replication, Holliday junctions, homologous recombination, NBS1

## Abstract

The MUS81 protein belongs to a conserved family of DNA structure-specific nucleases that play important roles in DNA replication and repair. Inactivation of the *Mus81* gene in mice has no major deleterious consequences for embryonic development, although cancer susceptibility has been reported. We have investigated the role of MUS81 in human cells by acutely depleting the protein using shRNAs. We found that MUS81 depletion from human fibroblasts leads to accumulation of ssDNA and a constitutive DNA damage response that ultimately activates cellular senescence. Moreover, we show that MUS81 is required for efficient replication fork progression during an unperturbed S-phase, and for recovery of productive replication following replication stalling. These results demonstrate essential roles for the MUS81 nuclease in maintenance of replication fork integrity.

## INTRODUCTION

Faithful DNA replication is essential for the maintenance of genome stability in all organisms. The ability of cells to minimize transmission of errors arising during DNA replication to daughter cells depends not only on dedicated DNA replication factors, but also on a large number of DNA repair/DNA damage tolerance proteins. These genome maintenance proteins can act at a number of different locations during the replication process, including upstream of the advancing fork, within the replisome itself, or ‘post-replicatively’ behind the fork. Amongst the many replication fork repair factors are those that serve to stabilize, process or cleave replication forks stalled either by dNTP exhaustion [1, 2] or by an encounter with lesions/adducts in the template DNA [3, 4]. Failure to execute genome duplication in a timely or accurate manner can generate a cellular state often termed ‘replicative stress’. This state is associated with the accumulation of markers of replication-associated DNA damage responses [5]. Importantly, in recent years it has become clear that replicative stress plays a key role during tumorigenesis [6–8]. Moreover, many solid cancers display evidence of persistent replicative stress [9, 10], and this phenotype is now widely considered to be an Achilles’ heel of tumor cells than can be exploited therapeutically [11, 12].

One process that plays a critical role in restoration of productive DNA synthesis at sites of stalled or collapsed replication forks is the homologous recombination repair (HRR) pathway [13]. HRR serves to restore replication following replication fork collapse after the fork encounters a ssDNA nick in the leading strand template, often through a process known as break-induced replication (BIR) [14]. BIR is implicated in driving some cancer-associated events in that it can induce genomic duplications in human cells. HRR can also act post-replicatively to fill ssDNA gaps that arise following replicative bypass of lesions on the lagging strand template. Interestingly, some of the factors that play a role in HRR also act directly on stalled replication forks outside the strict context of the HRR pathway. In human cells, these proteins include the MUS81 nuclease, the BLM helicase and a series of Fanconi anaemia pathway proteins. MUS81 is an evolutionarily conserved endonuclease that forms a complex with conserved interacting partners: Eme1 (in fission yeast) and Mms4 (in budding yeast) or either of the human EME1 and EME2 proteins [15]. These heterodimeric complexes are dual acting in yeasts, being involved both in resolving Holliday junction (HJ) structures during HRR [16, 17], and in processing stalled or otherwise disrupted replication forks [18, 19]. The human MUS81-EME1 and MUS81-EME2 complexes seem to function via a similar mechanism to their yeast orthologs [20, 21]. Mutation of either *mus81^+^* or *eme1^+^* in fission yeast causes very pronounced meiotic defects [17], although the phenotype is somewhat less severe in budding yeast. However, both budding and fission yeast *mus81* mutants are sensitive to DNA replication perturbing agents, such as UV irradiation, methylmethane sulfonate (MMS), hydroxyurea (HU), and camptothecin (CPT) [19, 22–24], suggesting an important role of Mus81 and its partners in some aspect of replication fork repair. Surprisingly, however, *Mus81^−/−^* mice are viable and do not show obvious defects, apart from increased tumor incidence [25–27], despite the fact that embryo fibroblasts from these knockout mice accumulate chromosomal aberrations [25]. Nevertheless, Mus81 is known to be required for generation of double strand breaks (DSBs) following replication stalling in mouse embryonic stem (ES) cells, in a process that is probably required for re-initiation of productive replication [28] and cleavage of common fragile sites during mitosis [29, 30].

In order to investigate the phenotypic effect of acutely disabling a putative HJ resolvase and replication fork processing factor in human cells, and to analyze the subsequent cellular responses to replication stress, we have analyzed human cells in which MUS81 was depleted using shRNA. We demonstrate a critical role for MUS81 in maintaining DNA replication fork progression. Furthermore, spontaneous replication lesions caused by loss of MUS81 induce a DNA damage response, cell cycle arrest and subsequent cellular senescence.

## RESULTS

### Depletion of MUS81 leads to cell cycle arrest and senescence

**Figure 1:**
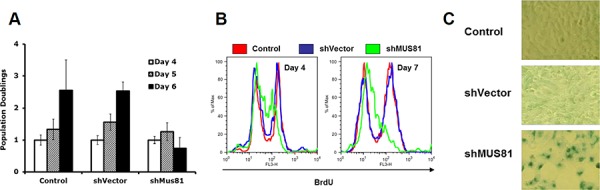
Depletion of MUS81 leads to proliferation arrest and cellular senescence **A.** MUS81 depleted U2OS cells fail to proliferate further after 5 days of viral infection. The averages and standard deviations (error bars) from at least three experiments are depicted. **B.** Flow cytometric analysis showing a dramatic drop in the BrdU positive staining (replicating) cell population following depletion of MUS81. **C.** MUS81-depleted cells undergo senescence after 7 days of viral infection, as indicated by staining for senescence-associated β-galactosidase (blue).

**Figure 2:**
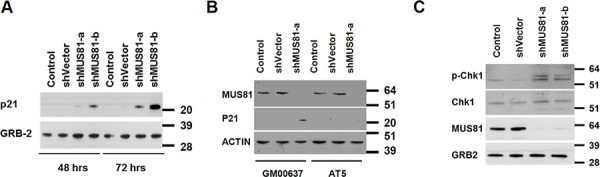
A checkpoint activation is seen in MUS81-depleted cells **A.** Western blot analysis of p21 and GRB2 protein levels after shRNA depletion of MUS81. Molecular masses are given in kDa. **B.** MUS81 depletion-mediated p21 induction is dependent on ATM. The AT5 cell line was derived from a patient with ataxia telangiectasia lacking ATM. Molecular masses are given in kDa. **C.** Western blot analysis of phospho-CHK1 (S317) and total CHK1 levels 3 days after shRNA depletion of MUS81. GRB2 is a loading control. Molecular masses are given in kDa.

To investigate a possible role for MUS81 in protecting DNA replication forks from irreversible breakdown, we used a previously validated adenoviral shRNA strategy to acutely deplete MUS81 from human U2OS cells [29]. Over a prolonged period of MUS81 depletion (4–7 days), the MUS81 shRNA-expressing cells showed significant phenotypes, including a profound proliferation defect (Figure 1A). Specifically, we observed that these cells began to undergo cell cycle arrest following 4 days of lentiviral infection (Figure 1B) and became senescent within 7 days (Figure 1C). This process was accompanied by ATM-dependent up-regulation of the senescence-associated factor, p21 (Figure 2A and 2B). These experiments were performed using either of 2 independent shRNAs, and the extent of depletion of MUS81 was >90% in these cells within 48–72 hours of virus infection (Figure 2B and 2C). To analyze whether other features of a putative cell cycle checkpoint activation were evident in the MUS81-depeleted cells, we studied activation of the CHK1 kinase, a defined marker of replication stress. We observed an increase in CHK1 phosphorylation following MUS81-depletion (Figure 2C), consistent with the presence of spontaneous DNA damage/replication stress in these cells.

### A DNA damage response is triggered after depletion of MUS81

**Figure 3:**
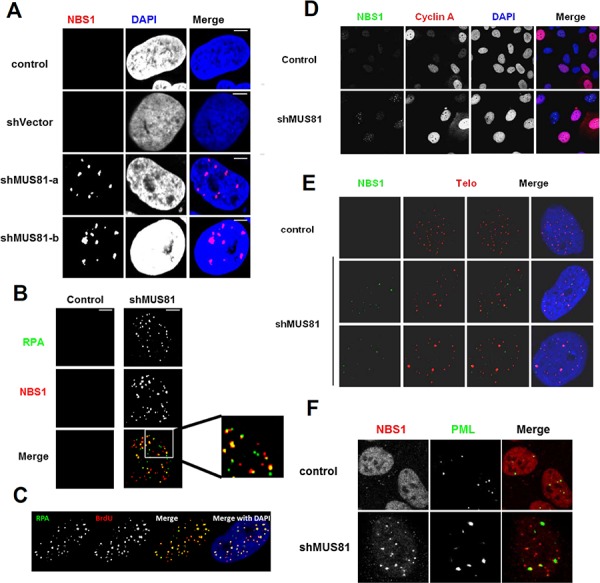
Loss of MUS81 activates DNA damage response **A.** MUS81 depleted U2OS cells accumulate NBS1 foci defining sites of DNA damage. **B.** Partial co-localization of NBS1 foci and RPA foci in MUS81-depleted cells. **C.** Co-localization of RPA foci to ssDNA regions. Cells were labelled with BrdU for 3 days concomitant with MUS81 depletion. BrdU was detected at ssDNA regions by immunofluorescence. **D.** NBS1 foci arise in cells that are both positive and negative for Cyclin A expression. Scale bars, 5 μm. **E.** NBS1 foci form largely at non-telomeric loci. **F.** Partial co-localization of NBS1 foci with PML nuclear bodies.

It has been reported that oncogene-induced senescence can occur via the activation of the DNA damage response triggered by persistent replicative stress [6, 31, 32]. To analyze whether the cellular senescence that we observed following depletion of MUS81 might also be due to DNA damage responses, we stained cells with antibodies against either NBS1 (a component of MRE11-RAD50-NBS1 (MRN) complex) or the ssDNA binding protein, replication protein A (RPA). Multiple nuclear foci representing both NBS1 and RPA were evident in nearly 90% of MUS81-depleted cells (Figure 3A and 3B), indicative of a constitutive DNA damage response. About 40% NBS1 foci were shown to co-localize with RPA staining. To address whether the RPA foci represent regions of ssDNA, the MUS81-depleted cells were incubated in medium containing BrdU, and then stained with an antibody recognizing BrdU. This antibody is specific for BrdU in ssDNA, and hence no denaturation of the DNA is required to reveal BrdU incorporation. We found that the RPA foci strongly co-localized with BrdU positive foci (Figure 3C), indicating that the RPA foci almost exclusively define ssDNA regions. To investigate if the NBS1 foci are seen only during a specific cell cycle phase, Cyclin A protein was analyzed by immunofluorescence in order to specifically define S/G2 phase cells. We observed that NBS1 foci were present in the MUS81-depleted cells regardless of their Cyclin A status (Figure 3D), suggesting that, even if these foci arise during a perturbed S-phase, they can persist in cells outside of S-phase.

It has been suggested that MUS81 may be involved in mediating telomeric recombination in ALT cells such as U2OS [33]. To address whether telomeres could be the source of a DNA damage signal that induces the formation of NBS1 foci, telomeres were labelled using FISH in the MUS81-depleted U2OS cells. We observed that the NBS1 foci only rarely co-localized with telomeres, suggesting that the damage signal mainly originates from non-telomeric loci (Figure 3E). We also asked if the NBS1 foci seen in MUS81-depleted cells co-localize with PML protein. We found that nearly all PML bodies co-localized with NBS1 foci (Figure 3F). However, because there were, on average, far fewer PML bodies per cell than NBS1 foci, the majority of NBS1 foci were not associated with a PML body.

### MUS81 plays important roles in replication fork progression

**Figure 4:**
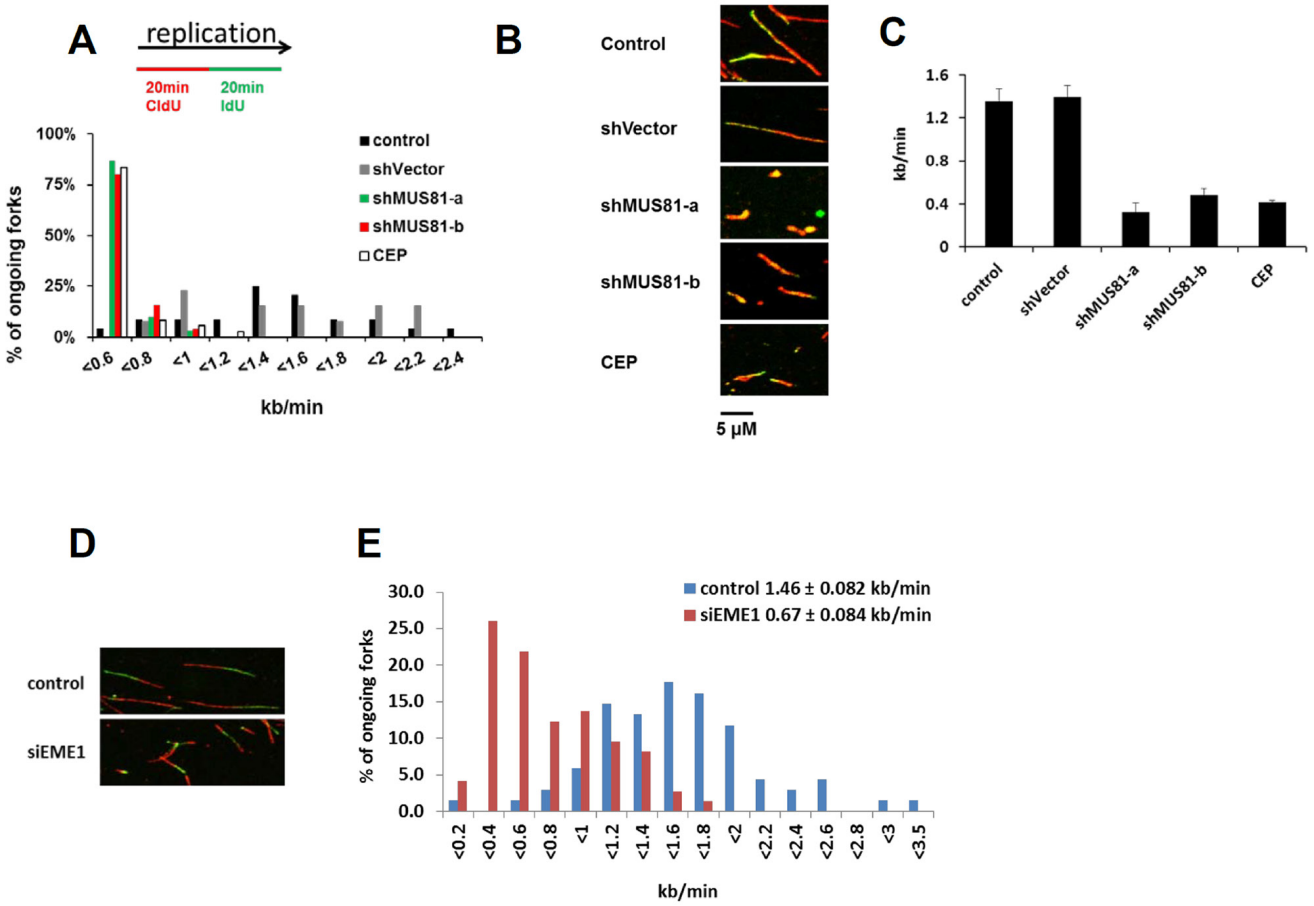
MUS81/EME1 controls replication progression speed **A.** Following any indicated treatment, cells were pulse-labelled with CIdU and IdU for 20 min each. Distribution of replication fork speeds in cells treated as indicated. **B.** Representative images of replication tracks from cells expressing no shRNA or the MUS81 shRNA for 3 days. Also shown are cells exposed to the Chk1 inhibitor, CEP-3891, for 2 hours. **C.** The average replication speed at indicated conditions and standard deviations from at least three experiments **D.** Representative images of replication tracks from cells depleted with EME1 siRNA for 3 days. **E.** Distribution of replication fork speeds in cells following indicated treatment. The average speed and standard deviations from at least three experiments were shown on the top right.

One possible explanation for our results is that spontaneous lesions arising during DNA replication in the absence of MUS81 generate a DNA damage response, leading ultimately to cellular senescence. Consistent with this proposal, yeast *mus81* mutants are hypersensitive to replication inhibiting agents [19, 22–24] and MUS81 has been shown to be involved in replication restart in mouse ES cells [28] as well as in resolution of replication stress-induced common fragile site expression [29]. To investigate whether MUS81-depleted human cells show inherent problems during an unperturbed S-phase, or only a failure to efficiently restart stalled forks, we utilized the DNA fibre assay to visualize the progression of individual replication forks. We quantified replication fork progression in MUS81-depleted U2OS cells taken for analysis in the early stages of viral infection (3 days) before any signs of cellular senescence were evident. Moreover, any cells that had ceased proliferation would not incorporate the nucleoside analogues used to mark sites of ongoing replication on the fibres and would, therefore, be eliminated from the analysis. Strikingly, we found that DNA replication fork speed was significantly reduced in the MUS81-depleted cells, being reduced to a level comparable to that of cells treated with an inhibitor of the CHK1 kinase (a known regulator of fork speed [2]) (Figure 4A–4C), indicating a fundamental requirement of MUS81 in DNA replication fork progression. Because this finding was surprising, given that MUS81 is a nuclease, and because of the possibility of potential off-target effects of the MUS81 shRNAs, we tested whether depletion of the MUS81 partner protein, EME1, also affected replication fork speed. We found that EME1 depletion also reduced fork speed significantly (Figure 4D and 4E), providing strong evidence that the effects on replication that we report are due to loss of MUS81-EME1 function and not to some off-target effect.

## DISCUSSION

Using shRNA-mediated depletion, we have demonstrated that Mus81-depleted human cells display multiple abnormalities in DNA metabolism. These defects ultimately trigger a constitutive DNA damage response, which we propose leads to MUS81-depleted cells undergoing cellular senescence. We report several abnormalities induced by depletion of MUS81 in U2OS cells. First, the cells accumulate multiple NBS1 and RPA foci, the latter of which form at sites of ssDNA accumulation, indicative of a DNA damage response. This occurs within 48 hours of exposure of the cells to *MUS81* shRNA. At later times (5–7 days) the cells cease proliferation and show classical features of cellular senescence as might be expected because MUS81 is required for the maintenance of telomeres in cells such as U2OS that lack telomerase [33]. However, strikingly, the MUS81-depleted cells show abnormalities during S-phase that are genome-wide and not telomere specific. Replication fork progression is significantly impaired even in ‘undamaged’ cells. Perhaps surprisingly, the defect in constitutive replication fork movement is as severe as that seen in cells exposed to an inhibitor of the CHK1 kinase, a known regulator of fork speed [2].

Previous studies on *Mus81^−/−^* mice have suggested that loss of Mus81 function does not markedly affect embryonic development. Moreover, the adult mice are phenotypically quite normal, with the exception of an increase in the frequency of sporadic tumours [25, 26]. These results appear at odds with our findings using acute depletion of MUS81 in human cells in culture. Although the function of mouse and human MUS81 may not completely overlap [34], there are several possible explanations for this apparent discrepancy that we have considered. First, and most obviously, our data might reflect on off-target effect of the *MUS81* shRNA that leads to depletion of another cellular factor. However, we consider this very unlikely, given that we see very similar effects using 2 independent shRNAs that share no sequence similarity or any obvious common target other than *MUS81*. Consistent with our interpretation, we previously reported that MUS81 depletion using these sequences suppressed SCEs in BS cells [29], as was reported by Wechsler et al., who used a different MUS81 shRNA sequence [35], and depletion of the MUS81 partner protein, EME1, also affected replication progression. A second possible explanation for our data is that the human cells used in our study show an intrinsically different response to replicative stress induced by MUS81 depletion than do mouse cells. This is possible, but it should be noted that MUS81 is highly conserved and plays roles in genome maintenance in many species, including yeasts. Our preferred explanation is that a mechanism exists for *Mus81^−/−^* mouse cells to ‘adapt’ to the loss of Mus81 protein over time that isn't revealed when the protein is acutely depleted. It may be significant in this regard that targeting frequencies for the *Mus81* locus in mouse ES cells are very low [25]. This poor targeting efficiency may reflect a low frequency of genetic or epigenetic suppression of the *Mus81* defect that permits rare survivors of the targeting regimen to be recovered.

The most striking effect of MUS81 depletion is to cause a reduction in the rate of replication fork progression, a finding also observed following depletion of EME1. Hence, we must consider how a nuclease such as MUS81 might affect replication fork progression. It seems very unlikely that MUS81 plays a direct role in DNA synthesis *per se*. Instead, we favour the view that certain aspects of natural fork movement through a DNA double helix might create a periodic requirement for MUS81 to act. One possibility is that MUS81 is required to reactivate forks that have stalled for some reason. A second, related possibility is that MUS81 acts at sites where an atypical DNA structure has arisen during replication fork progression. Given that MUS81 can cleave 4-way DNA structures resembling Holliday junctions, it is possible that a failure to have prompt relief of torsional stress ahead of the advancing replication fork causes the fork to periodically reverse (so-called fork regression), generating a 4-way DNA junction. Indeed, recent data indicate that fork regression is a remarkably frequent event in human cells [36]. MUS81 might be required to cleave such regressed forks in order to permit restoration of normal fork progression. If this proposal is relevant to the phenotype we have observed by chromosome fiber analysis, the underlying problem must occur quite frequently in the genome during S-phase because the average fork speed was consistently reduced in the multiple fibers that we analyzed. A further possibility is that, at least under some circumstances, MUS81 itself acts to relieve the torsional stress induced by the replication fork movement. Although, this role is primarily performed by certain DNA topoisomerases [37], some situations might necessitate the ssDNA-nicking activity of MUS81 to relieve the stress.

We propose a model in order to explain the observed senescence induced following MUS81 depletion. We suggest that reduced replication fork progression in MUS81-depleted cells result in accumulation of replication-associated ssDNA. Such unresolved replication structures are the likely source of the signal for the activation of the ATR-Chk1 DNA damage response pathway, mediated through ATRIP binding to RPA [38]. It is well established that extended DNA damage signaling following replication stress can induce cellular senescence [6, 31, 32].

A previous study [33] reported, as we do, that MUS81 depletion from human ALT cells leads to growth arrest. We have extended this to show that the cells undergo replicative senescence that is accompanied by a strong DNA damage response. The most straightforward explanation for this would be that telomere instability elicits a signal that activates the formation of foci containing DNA damage/repair factors. However, we found only a modest degree of co-localization between the foci containing NBS1 and RPA, and sites of telomeric stress. Coupled with the fact that we have defined a general defect in replication fork progression in MUS81-depleted cells, we suggest that telomeres cannot be the sole source of the DNA damage signal that induces growth arrest in cells with impaired MUS81 function.

In summary, we have shown that acute and severe depletion of MUS81 from human cells in culture elicits a constitutive DNA damage response that is associated with intrinsic defects in DNA replication fork progression and ultimately leads to cellular senescence. A plethora of cell cycle checkpoint and DNA repair mechanisms have evolved in order to safeguard genome integrity in a coordinated fashion with DNA replication. Loss of any of these mechanisms promotes the development of ‘chromosome instability’ disorders, and often an increased risk of cancer [39]. Mus81^*−*/*−*^ mice have a predisposition to the development of spontaneous lymphomas, breast cancer and prostate cancer compared to haploinsufficient Mus81 mice [26, 27, 40]. Moreover, Mus81^*−*/*−*^ p53−/− double mutant mice develop excess sarcomas [27] and Blm−/− Mus81−/− double mutant mice show significantly increased risk of tumour development compared with single mutants [41]. Reduced MUS81 expression has also been observed in human carcinomas including hepatocellular carcinoma [42] and colon carcinoma [43], and this is associated with poor prognosis. Moroever, single nucleotide polymorphisms in human MUS81 that reduce protein activity are a proposed breast cancer susceptibility factor [44]. Taken together, these data indicate that *MUS81* is a tumor suppressor gene and plays a role in suppressing the development of different malignancies [25–27, 40].

## MATERIALS AND METHODS

### Cell culture and RNA interference

Normal human fibroblasts, GM00637 and ATM-deficient fibroblasts (AT5) cells were grown in Minimum Essential Medium (MEM) Alpha Medium, while U2OS cells were grown in Dulbecco's modified Eagle's medium (DMEM) with 10% fetal bovine serum at 37°C under an atmosphere containing 5% CO_2_. The Chk1 inhibitor CEP-3891 was provided by Cephalon Inc., and was used at a concentration of 500 nM, as described previously [2]. To deplete human MUS81, the following shRNAs were used: shMUS81-a (5′-GAGTTGGTACTGGATCACATT-3′); shMUS81-b (5′-CCTAATGGTCACCACTTCTTA-3′). Pooled GeneSolution siRNA against EME1 was purchased from Qiagen (5′-CACAGCCA GTCAGGTTGCTAA-3′; 5′-GAGGAGTGCAGCAGATAACAA-3′; 5′-CAGAATTTGCTC GCAGACATA-3′; 5′-AAGGACCTGATCTTAGATCCA-3′).

### Immunofluorescence and FISH

Following any indicated treatment, cells were fixed and stained as described previously [45]. The primary antibodies used were against NBS1 (1:1000, NB100–143, Novus), RPA (1:1000, ab2175, Abcam), PML (1:1000, ab94471, Abcam) and Cyclin A (1:200, sc-56299, Santa Cruz). The secondary antibodies were AlexaFluor 488 or 555-conjugated goat anti-rabbit or anti-mouse IgG (1:500, all from Life Technologies).

For combined immunofluorescence and FISH, samples were exposed to the appropriate primary and secondary antibodies (as above) and subjected to re-fixation in paraformaldehyde (8%) at 4°C for 20 min. A telomere PNA FISH kit (DakoCytomation) was used to detect telomeres. Samples were denatured at 80°C for 3 min under a coverslip in the presence of the Cy3-conjugated PNA probe. Slides were then hybridized in the dark at room temperature for 30 min, followed by a brief rinse and a post-hybridization wash with a wash solution at 65°C for 5 min. DNA was counterstained with DAPI.

### Western blotting

Cells were lysed in RIPA buffer in the presence of 1 × protease inhibitor cocktail (Sigma). An aliquot of 50 μg total protein was run on an SDS-PAGE gel and transferred to Hybond ECL membrane (GE Healthcare). This membrane was immuno-blotted with antibodies against MUS81 (1:1000, ab14387, Abcam), GRB2 (1:1000, 610112, BD Transduction Laboratories), p21 (1:1000, 2947, Cell Signaling Technologies), phospho-CHK1 (pS317, 1:1000, A300–163A, Bethyl), CHK1 (1:1000, sc-7898, Santa Cruz), and Actin (1:5000, A2547, Sigma). Immuno-reactive proteins were visualized using ECL reagents (Roche) following the manufacturer's instructions.

### Cell proliferation assays

Cells were infected with adenoviruses carrying the indicated shRNA in a multiwell plate, and the cell proliferation was measured as described elsewhere [46, 47]. Each day 4 after infection, cells in one well were counted with a haemocytometer.

### FACS analysis

Cells were pulse-labelled with BrdU (20 μM) for 30 min before harvesting. Following fixation in 70% ethanol and denaturation in HCl (2 M), cells were stained with a mouse monoclonal antibody against BrdU (BD Biosciences). After staining with a fluorescence-conjugated secondary antibody, cells were analysed using a FACSCalibur Flow Cytometer (Becton Dickinson).

### Measurement of cellular senescence

Cellular senescence was determined using a Senescence Staining Kit (Cell Signaling Technologies) following instructions provided by the manufacturer. Following the indicated treatments, cells were fixed and stained for the presence of β-Galactosidase using the staining solution supplied. The solution was removed when a blue colour had developed and 70% glycerol was added for long-term sample storage.

### DNA Fibre assays

Cells were pulse-labeled with 25 μM CIdU for 20 min, washed 3 times with medium, and then incubated in 2 mM HU for 2 hours. After washing with medium, 250 μM IdU was added for 20 min. Labelled cells were harvested and DNA fibre spreads were prepared as described previously [48]. For immunodetection of CIdU-labelled tracts, acid-treated fibre spreads were incubated with a rat anti-BrdU monoclonal antibody (AbD Serotec) that recognises CIdU, but not IdU, for 1 hour at room temperature. Slides were fixed with 4% formaldehyde, and were then incubated with an AlexaFluor 555-conjugated goat anti-rat IgG (Life Technologies) for 1.5 hour at room temperature. IdU-labelled patches were detected using a mouse anti-BrdU monoclonal antibody (Becton Dickinson) that recognises IdU, but not CIdU, by incubation overnight at 4°C, followed by incubation with an AlexaFluor 488-conjugated goat anti-mouse F(ab’)_2_ fragment (Life Techologies) for 1.5 hour at room temperature. Fibres were examined using a using a Biorad Radiance confocal microscope using a 60 × (1.3 NA) lens. The lengths of CIdU (AF 555, red) and IdU (AF 488, green) labelled tracts were measured using the ImageJ software, and values of tract length were converted into DNA kilobases using the conversion factor 1 μm = 2.59 kb [48]. Replication structures were quantified using the Cell Counter Plug-in for ImageJ (Kurt De Vos, University of Sheffield, UK).
